# Feasibility of Using Pulsed Electromagnetic Field Therapy to Improve the Dynamic Postural Balance of Children with Cerebral Palsy: A Randomized, Sham-Controlled Pilot Study

**DOI:** 10.3390/jcm14010192

**Published:** 2024-12-31

**Authors:** Márk Ágoston Pulay, Krisztina Kornis, Gabriella Bednárikné Dörnyei, Éva Feketéné Szabó, Mónika Horváth, Attila Matiscsák, Csaba Nyakas, Andrea Tenk Miklósné Zsebe, Tímea Vissi, Ágnes Mayer, Ibolya Túri

**Affiliations:** 1 Pető András Faculty, Semmelweis University, 1125 Budapest, Hungary; 2 Department of Ergonomics and Psychology, Faculty of Economic and Social Sciences, Budapest University of Technology and Economics, 1111 Budapest, Hungary; 3 Faculty of Health Sciences, Semmelweis University, 1085 Budapest, Hungary

**Keywords:** cerebral palsy, pulsed electromagnetic field therapy, balance, trunk control

## Abstract

Cerebral palsy (CP) manifests with abnormal posture and impaired selective motor control, notably affecting trunk control and dynamic balance coordination, leading to inadequate postural control. Previous research has indicated the benefits of pulsed electromagnetic field (PEMF) therapy for various musculoskeletal and neurological conditions. Therefore, we conducted a randomized pilot study to assess the feasibility of our preliminary research design and examine the effect of the PEMF treatment among children with CP. **Methods**: Twelve children with spastic CP participated, with the study group undergoing PEMF treatment three times a week for four weeks. The treatment involved sine signal form, 20/200 Hz frequencies at an amplitude of 150 μT, initially administered for 8, 12, and 16 min per session. The control group received a sham treatment. Dynamic postural balance was evaluated using a force platform at baseline and post-intervention, and the data were analyzed. Data were processed using IBM SPSS 27 by repeated factorial analysis of variance. The significance level was α = 0.05. **Results**: No side effects of PEMF therapy were detected; this is important, because this intervention has not yet been applied among CP patients. The treatment group demonstrated a positive trend in fine balance coordination tests (*p* = 0.049); however, the small sample size and variability in control group performance suggest caution in interpreting these findings. Other test domains did not show significant differences. **Conclusions**: Our pilot study reveals the safety, feasibility, and potential efficacy of pulsed electromagnetic field (PEMF) therapy for children with cerebral palsy. With no observed side effects, the significant improvement in fine balance coordination suggests a promising avenue.

## 1. Introduction

Cerebral palsy (CP) is the most common condition requiring complex rehabilitation in childhood. The prevalence of pre-/perinatal CP in high-income countries is 1.5/1000 live births [1]. Its most common triggers include premature birth, fetal growth restriction, genetic factors, and pregnancy infection [2]. CP is an umbrella term, and types are based on the primary motor disorder: spastic, dyskinetic, ataxic, or mixed. Spastic CP is the most dominant type of CP, with prevalence rates of approximately 70–86% [3,4,5,6]. Children with CP have several functional limitations in fine motor skills (grasping, writing), gross motor functions (crawling, sitting, walking, and transfer activities), and other related functional mobility tasks in activities of daily living (ADL) [7]. CP is characterized by abnormal posture and a lack of selective motor control, such as poor trunk control and dynamic balance coordination, which contribute to insufficient postural control and considerable limits in everyday activities. Balance and upright postural control are essential to maintaining body posture in a sensory environment with anticipatory and automatic postural adjustments [8]. The association between upper extremity function and trunk control has also been proved [9].

Several pharmacological and surgical methods are available for the symptomatic treatment of CP [10]. Among the physiotherapy interventions, intensive activity-based, goal-directed interventions, gait training, and constraint-induced movement therapy (CIMT) are effective in functional improvement [11,12]. Among physical agents, there is only moderate evidence for the effectiveness of functional electrical stimulation (FES) in improving muscle strength, range of motion, and function in children with CP [11].

Pulsed electromagnetic field (PEMF) therapy interacts with biological systems by modulating ion transport and binding at cell membranes, promoting tissue repair and cellular signaling. Unlike continuous electromagnetic fields (EMFs), PEMF uses pulsed signals to target stressed or injured tissues, enhancing their response. This makes PEMF particularly relevant for conditions, like cerebral palsy (CP), where motor impairments and disrupted homeostasis are common, though its effects in CP require further investigation [13].

PEMF has been shown to influence intracellular calcium levels, nitric oxide (NO) production, and free radical processes, which contribute to improved microcirculation and reduced inflammation—factors critical for managing CP symptoms [14]. Additionally, PEMF has been linked to neuroplasticity by affecting growth factors, as demonstrated in stroke models [15]. Its anti-inflammatory effects include the reduction of cytokines, like TNF-alpha and IL-6, along with enhancements in osteogenesis and chondrocyte proliferation [16].

Effective PEMF therapy utilizes low-frequency electromagnetic currents with a broad range of frequencies, designed to enhance cell membrane permeability and stimulate various intracellular functions. The therapy’s ability to penetrate tissues deeply and activate proprioceptive sensory fibers underscores its potential as a non-invasive tool for spasticity management in CP. While transcranial magnetic stimulation (TMS) has been extensively studied for brain-focused spasticity management, peripheral applications of PEMF remain underexplored. Nevertheless, its potential to improve neuromuscular tissue function, enhance blood perfusion, and exert anti-spastic effects makes PEMF a promising avenue for further research in CP populations [17].

PEMF has been frequently used as a non-invasive additional treatment for patients with different kinds of musculoskeletal disorders (MSDs), like arthritis, abnormal muscular tone (sclerosis multiplex, muscular dystrophies), and osteoporosis [18,19]. Most of the studies focused on the possible effect of pain reduction in different spinal or back pathologies, such as lower back pain, disc herniation, spinal lesions, or even after knee arthroplasty [20,21,22,23].

Richards et al. conducted a clinical trial among patients with multiple sclerosis (MS), and they found significant improvement in the performance scale combined rating for mobility, spasticity, and vision between the intervention and placebo groups [24]. Yadollahpour and Rashidi investigated the therapeutic applications of EMFs in MSDs; they found that EMF stimulations have therapeutic benefits for different MSDs [18]. A couple of studies published findings of a decrease in spastic tone and joint movements after repetitive PEMF therapy. Despite these promising findings, PEMF has not yet been established as a standard clinical therapy due to inconsistent evidence from studies with varying intensities, frequencies, and methodological quality [22,23,25].

To date, no studies have specifically investigated the potential benefits of PEMF therapy in individuals with CP, highlighting a critical gap in the literature. This pilot randomized controlled trial was, therefore, designed to address this gap. The primary objectives were to evaluate the feasibility of the research design and investigate a standardized dosing protocol for PEMF therapy. Additionally, the study aimed to assess the effects of the intervention on the dynamic postural balance of children with CP.

## 2. Materials and Methods

Children with cerebral palsy are a highly heterogeneous population with a wide range of movement abilities [26]. The large number of studies with small sample sizes reflects the challenges that researchers face in recruiting a large and homogenous sample from this population due to the high clinical variability of CP [27].

Participants in this study were recruited by personal request among the children at the Conductive Primary School at András Pető Faculty, Semmelweis University, Hungary.

The children were eligible for inclusion if they met the following criteria:

- Between 6–12 years.
- Diagnosed with spastic CP.
- GMFCS level I to IV (able to sit in the chair, able to control their trunk, head, and limb movement).

Children were excluded from the study based on the following criteria:

- Participants who had received botulinum toxin injections or undergone orthopedic surgery in the previous six months were excluded to avoid interference with other therapies.
- Participants with any history of epilepsy were excluded from the study to ensure safety. The potential risks associated with an epileptic episode outweigh the expected therapeutic benefits of the intervention. This precautionary approach was taken to prioritize participant safety.

### 2.1. Participants

Twelve participants (female 41.67%–male 58.33%, average age of nine years two months) were involved in the study; 75% of the involved children were diagnosed spastic tetraparesis and 25% were diagnosed spastic hemiparesis. Regarding GMFCS, 25% were at level I, 16.67% were at level II, 25% were at level III, and 33.33% were at level IV. The characteristics of the participants are shown in Table 1.

### 2.2. Study Design

**Figure 1:**
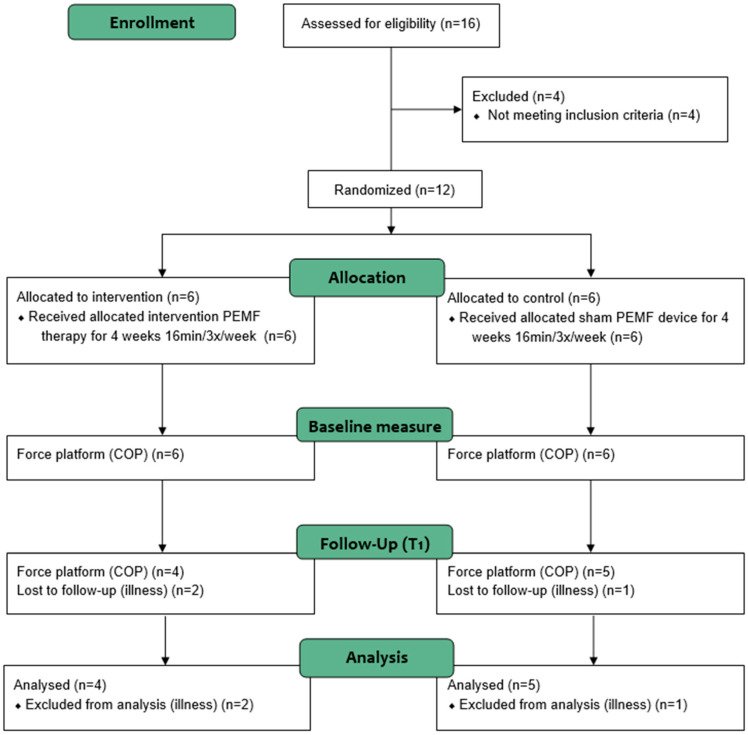
Flowchart of the study design.

Patients who met the inclusion criteria were randomly divided into two groups, in a 1:1 ratio, based on a computer-generated randomization process. The classification key was revealed to everyone participating in the research at the end of the study period. The experimental design of the study is shown in Figure 1.

### 2.3. Intervention: PEMF Device

**Figure 2:**
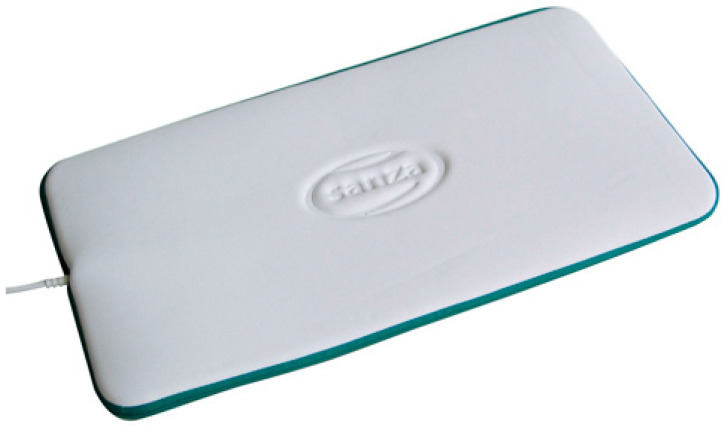
Sanza cushion applicator.

The PEMF device used in this study (Sanza Profi system CRM Cell-Regeneration GmbH, Salzburg, Austria) is a therapeutic device that delivers a pulsed electromagnetic field (intensity of 10–300 µT) at different frequencies. Different PEMF applicators are available, such as mat (whole-body applicator), which uses lower (brainwave) frequencies and simultaneously influences all of the cells in the body, as well as local applicators, such as cushions, which have an additional acute frequency range. We used the cushion applicator (Figure 2), whose shape is ideal for treating large muscles, such as the limbs and trunk.

The intensity of the magnetic field refers to the amplitude of the signals—the range of extension. The special programs of the Sanza device primarily use the highest intensity settings, and the selected waveform and the frequency influence this parameter. Frequency (range 1–200 Hz) refers to how often the signal cycles per second, and the waveform relates to the shape of the signals (sine, sawtooth, inverse sawtooth, and rectangle). Based on the device guidelines, during the intervention in the current pilot study, we started with a basic and low-intensity program (20/200 Hz and 150 µT amplitude), in the first week, three times for 8 min each; in the second week we employed a treatment of three times for 12 min each, while the last two weeks, the treatment was carried out three times for 16 min each. During the PEMF therapy application period, the participants were asked to sit, and the cushion applicator was placed on their lap, with both of their hands on the instrument. This body position can allow the local cushion applicator to affect the subjects’ limbs and trunk. The control group used a sham device that was externally identical to the active PEMF device but deactivated, ensuring that no electromagnetic field energy was generated. Participants were unaware of the sham treatment, maintaining blinding to group allocation. The study implemented a single-blind design for participants and a partial double-blind protocol for researchers. Conventional therapy providers and the personnel recording the measurements were also blinded to group assignments. Only the individual administering the PEMF therapy was aware of the group allocation to ensure the proper delivery of the intervention.

The planned indicator measurements were performed for each group at baseline and immediately after the intervention. Chronic effects were reassessed after the intervention period. Acute effects were assessed within 1 h of treatment/placebo treatment.

### 2.4. Measurement—Force Platform

A force platform was chosen as the sole measurement tool for this study due to its ability to precisely evaluate dynamic postural balance, a critical aspect of functional mobility in children with cerebral palsy (CP). Postural balance was prioritized, as it directly impacts activities of daily living and rehabilitation outcomes. Other potential measures, such as spasticity and pain, were excluded due to practical constraints. Spasticity measurements are most valid immediately after interventions, which was not feasible in this study, while pain was not deemed a primary concern in this young CP population. The force platform provided objective, quantitative data on postural adjustments, aligning closely with the study’s goals.

To evaluate the dynamic postural balance of the participants, we used a force platform (Bretz ZWE-PII), which consisted of a force platform (size: 0.5 m × 0.5 m × 0.1 m; range: 20–2000 N; linearity: ±1.5%; hysteresis: ±1.5%), amplifiers, a micro-computer, a PC, a monitor, and custom software. The center of pressure (COP) data were sampled at 100 Hz. A stool was placed on the force platform, and the participants sat on it with their feet resting parallel at hip-width on the platform and their palms resting on the stool next to their hip. The monitor was positioned at the subject’s eye level, and the hips, knees, and ankles were flexed at 90 degrees. Before taking measurements, each participant was given the opportunity to perform the exercise once as a practice. Subsequently, the results from the second measurement were recorded. To minimize learning effects and prevent fatigue, given the task’s requirement for intense concentration, only a single measurement was registered for analysis.

The assessment software contains two tasks (coordination tests 1 and 2) that require individual shifts of COP according to the task. These tasks are as follows:

- Coordination test 1 (“coloration test”): moving the COP within a given area. A 2 cm square was displayed in the middle of the screen. Subjects had to move the COP—which appeared as a pencil—inside the square to color in as much of the square as possible in 20 s and to minimize time spent outside the box. Variable 1. Coloration success: the size of the square’s coloration, expressed as a percentage of the total area of the square. Variable 2. Coloration time: time the COP spent in the square during the given time (20 sec) as a percentage (%).
- Coordination tests 2 (“Christmas tree test”): moving the COP to designated points. A Christmas tree with seven candies was displayed on the monitor. Subjects had to move the COP—which appeared on the monitor as a small circle—one by one to the sweets as quickly as possible. As soon as their COP covered all the targets, the figure disappeared from the screen, indicating that the mission had been successfully completed. Participants were given 20 s to complete this challenge. Variable: The number of candies reached within 20 s, expressed as a percentage of the number of available candies (7 pcs)—if a participant reached all the targets in 20 s, they achieved a score of 100%.

### 2.5. Data Collection

The demographic data and the GMFCS level were generated from the András Pető Faculty, Semmelweis University institutional database.

The research aimed to investigate the relationship between the treatments with the SANZA PEMF device (independent variable) and the measurements (dependent variable) performance, for which we collected quantitative data. We conducted an experimental study design. The measurement data show the performance of the subjects in the examined subtasks. The researchers recorded these data during the measurements, and all other relevant information was recorded, e.g., the necessary modifications due to the condition of the children. A digital database was then created from the results of each measurement. During the treatments, the researchers who conducted the treatments also kept a database of how many treatments the subjects received during the four weeks.

### 2.6. Data Analysis

Descriptive statistics including the mean, standard deviation, and percentage calculation were computed for all variables. A factorial repeated measure analysis of variance was performed to assess the effect of intervention and group differences, as follows:

- Within-subject factor (time): baseline vs. T1 (test and re-test after intervention period).
- Between subject factor (group): intervention and control.
- Dependent variables: coloration success %, coloration time %, and Christmas tree %.

Time main effect and time x group interactions were analyzed; the difference between baseline and T1 measurements by group was examined using simple main effects analysis. Additionally, percentage change was calculated for each dependent variable to examine the difference between the baseline and T1 using the following formula:

Change of mean in % = $\frac{T1-Baseline}{Baseline}\times 100$; a positive value is an increase, and a negative value is a decrease. The level of significance was set at α = 0.05. Data were processed using IBM SPSS Statistics for Windows, Version 27.0 (IBM Corp. Released 2017. Armonk, NY, USA: IBM Corp.).

## 3. Results

This study aimed to evaluate the feasibility and potential efficacy of PEMF therapy in children with CP, specifically focusing on its effects on dynamic postural balance. The intervention protocol, as described in the Methods section, was standardized to ensure consistency across participants, with baseline and post-intervention (T1) measurements taken for all assessed variables. The following results provide insights into the observed changes in dynamic postural coordination and the feasibility of implementing PEMF therapy in this population.

### 3.1. Comparison of Treatment and Control Group Outcomes

Table 2 shows the results of the simple main effects and differences between the baseline and T1 measurements by groups (treatment vs control). Three participants were not able to attend the T1 measurement. A significant change was found in dynamic balance coordination test 1 “Coloration success” (the size of the coloration of the square, expressed as a percentage of the total area of the square), with a mean percentage increase of 20.5% in the treatment group showing a strong effect size (F(1, 3) = 10.378, *p* = 0.049, η^2^_p_ = 0.78). A higher percentage was revealed at T1 than at baseline in the treatment group. The control group did not show positive changes (−25.4%) in this test item. Coloration time (time COP spends in the square during the 20 s in percentage) did not differ between the baseline and T1 measures (0.2%) in the treatment group. Still, there was a significant decrease, with a mean percentage decrease of 38% in the control group showing a strong effect size (F(1, 3) = 12.336, *p* = 0.025, η^2^_p_= 0.76). No statistically significant difference was measured in the Christmas tree test in the treatment (9.3%) or in the control group (−4.8) (*p* > 0.05).

Overall, the treatment group improved in two tests, while the control group worsened in two tests and showed a slight improvement in one test.

### 3.2. Safety Outcomes

No adverse events or side effects were reported during the PEMF intervention. This indicates that the applied dose and protocol were safe for the study population, supporting the feasibility of PEMF therapy in this clinical context.

## 4. Discussion

This pilot study investigated the feasibility and potential efficacy of pulsed electromagnetic field (PEMF) therapy in children with cerebral palsy (CP), focusing on dynamic postural balance. The intervention was well-tolerated, with no side effects observed, supporting the safety of the applied dose and protocol. Patients with CP often struggle with poor trunk control and dynamic balance coordination, which significantly limit their everyday activities and quality of life [8]. Therefore, our intervention targeted the trunk, hips, and thigh region. Dynamic postural coordination assessment (with a force platform) was performed based on visual information and visual feedback at the baseline and after the intervention period (T1). The evaluation contains two subdimensions. The success of the coordination test 1 (coloration success) significantly improved in the treatment group. The result of the coordination test 2 (Christmas tree) was also enhanced only in the treatment group. We presumably could not detect a significant change due to the ceiling effect (100%); those who achieved close to or exactly 100% in the first measurement could no longer improve. In the case of coloration success and time, the result of the control group worsened. This decline may reflect natural variability in motor performance or fatigue due to the study’s timeline. Additionally, outlier data from two control participants may have amplified the observed differences, underscoring the importance of larger sample sizes to mitigate the influence of individual variability.

Feasibility was a primary goal of this study, and our findings provide valuable insights. All participants adhered to the intervention schedule, and the absence of adverse events suggests that the protocol is both practical and safe for this population. However, the withdrawal of three participants before the final measurement highlights the challenges of long-term engagement in this population. Future studies should address this by improving participant retention strategies, such as closer communication with families and increased flexibility in scheduling sessions.

This study has several limitations that must be acknowledged. The small sample size restricts the generalizability of our findings and limits their statistical power, particularly for subgroup analyses. The lack of a chronic follow-up measurement is another limitation, as it prevents conclusions about the long-term effects of PEMF therapy. Additionally, while the force platform provided valuable data on dynamic postural balance, more comprehensive assessments, such as spasticity and muscle tone evaluations, could provide a fuller picture of PEMF’s impact.

Despite these limitations, this pilot study highlights key strengths, including the novel application of PEMF therapy in a CP population and the focus on dynamic balance—a critical but underexplored outcome. The results indicate a positive trend in treatment efficacy, supporting further investigation in a larger randomized controlled trial.

## 5. Conclusions

The aim of this pilot study with a relatively small number of patients was to examine the feasibility of the intervention setup, such as dosing, intensity, and body position, and the suitability of dynamic postural balance coordination test methods. Based on our initial results and experience, several modifications are planned in dosage and design, and additional efficacy tests will be performed.

Some significant shifts in dynamic postural balance coordination were observed in the treatment group, which is a good predictor of the direction of the planned RCT. In summarizing the results, it is necessary to note the limitations of the pilot study, such as the small number of subjects.

Based on these findings, several recommendations for future research have been formulated:

- Increase the sample size and implement a crossover study design to enhance the statistical power and reduce inter-subject variability.
- Extend the intervention period to at least eight weeks to assess both acute and long-term effects of PEMF therapy.
- Incorporate additional assessment tools, such as digital palpation devices, to evaluate changes in muscle tone, stiffness, and elasticity comprehensively.

The research limitations revealed during the pilot study and the research decisions formulated to address them will help us to properly prepare the crossover study, finalize the research focus, and refine the research methodology.

## Figures and Tables

**Table 1:** The characteristics of participants.

Age (Year)	Min-Max: 6–12	Average: 9.2
Gender	N	%
Female	5	41.67
Male	7	58.33
Diagnosis	N	%
Spastic hemiparesis	3	25
Spastic tetraparesis	9	75
GMFCS level	N	%
Level I.	3	25
Level II.	2	16.67
Level III.	3	25
Level IV.	4	33.33

**Table 2:** Comparison of the dynamic postural control measurement test (coloration success, coloration time, and Christmas tree) score groups.

	Treatment Group (*n* = 4)				
	Baseline	T1	F	*p*	η^2^_p_
Coloration success %, M(SD)	39 (2.5)	47 (6.7)	10.378	0.049	0.78
Coloration time %, M(SD)	95.5 (3.3)	95.3 (3.3)	0.022	0.893	0.01
Christmas tree %, M(SD)	91.5 (9.8)	100 (0)	3.000	0.182	0.50
	Control group (*n* = 5)				
	Baseline	T1	F	*p*	η^2^_p_
Coloration success %, M(SD)	44.8 (12.8)	33.4 (22.2)	1.271	0.323	0.24
Coloration time %, M(SD)	81.6 (17.4)	50.6 (26.7)	12.336	0.025	0.76
Christmas tree %, M(SD)	84 (29.1)	80 (21.7)	0.356	0.583	0.10

M: mean; SD: standard deviation; *p* < 0.05; F: F value; η^2^_p_: partial eta squared (η^2^_p_).

## Data Availability

The data that informed this article were not stored in a public database due to restrictions related to storing research data.
